# An evaluation of novel AMP2-coated electrospun composite scaffolds for intraoral bone regeneration: a proof-of-concept *in vivo* study

**DOI:** 10.3389/fbioe.2025.1443280

**Published:** 2025-04-17

**Authors:** Blaire V. Slavin, Shangtao Wu, Savanah R. Sturm, Kevin K. Hwang, Ricky Almada, Nicholas A. Mirsky, Vasudev Vivekanand Nayak, Lukasz Witek, Paulo G. Coelho

**Affiliations:** ^1^ University of Miami Miller School of Medicine, Miami, FL, United States; ^2^ Department of Biochemistry and Molecular Biology, University of Miami Miller School of Medicine, Miami, FL, United States; ^3^ Dr. John T. Macdonald Foundation Biomedical Nanotechnology Institute (BioNIUM), University of Miami, Miami, FL, United States; ^4^ Biomaterials and Regenerative Biology Division, NYU College of Dentistry, New York, NY, United States; ^5^ Hansjörg Wyss Department of Plastic Surgery, NYU Grossman School of Medicine, New York, NY, United States; ^6^ Department of Biomedical Engineering, NYU Tandon School of Engineering, Brooklyn, NY, United States; ^7^ DeWitt Daughtry Family Department of Surgery, Division of Plastic and Reconstructive Surgery, University of Miami Miller School of Medicine, Miami, FL, United States

**Keywords:** bone regeneration, *in vivo*, mandibular defect, electrospinning, AMP-2, composite scaffold, porcine xenograft

## Abstract

**Background:**

Alveolar ridge preservation by guided bone regeneration (GBR) is a surgical procedure that can be performed prior to implant placement to increase the likelihood of survival. Autogenic, allogenic, or xenogeneic derived bone (particulate graft) are frequently placed in conjunction with a barrier membrane for GBR; however, advancements in tissue engineering have led to the development of promising synthetic alternatives. Fiber-based scaffolds exhibit high surface-to-volume ratio and thereby improve cellular adhesion, reduce the likelihood of dehiscence and poor bone regeneration often associated with poorly immobilized particulate graft. This study aimed to evaluate the *in vivo* performance of a novel electrospun composite scaffold coated in a recombinant variant of human bone morphogenetic protein-2 (OsteoAdapt) relative to a porcine-derived xenograft. Further, it sought to determine if OsteoAdapt would remain within the defect without a membrane in place, as this is not feasible with the particulate xenograft currently used in clinical practice.

**Methods:**

Four-walled mandibular defects were created in each adult beagle dog (n = 4 defects per dog; n = 4 dogs for a total of 16 defects). Each defect received one of three experimental (test) groups: (i) OsteoAdapt without membrane (OA), (ii) OsteoAdapt with porcine membrane (OA/ZM), (iii) OsteoAdapt mixed with porcine particulate xenograft (Zcore™) with porcine membrane (OA/P/ZM) and compared to a positive control - Zcore™ with porcine membrane (CTRL). After 4-weeks *in vivo*, bone regeneration was assessed through qualitative volumetric reconstruction, qualitative and quantitative histological analyses.

**Results:**

Histomorphometric measurement of bone regeneration (% bone) within the region of interest revealed no significant differences between OA, OA/ZM, or OA/P/ZM in comparison to the CTRL at 4-weeks (*p* = 0.086, *p* = 0.218, and *p* = 0.806, respectively). Similarly, evaluation of soft tissue presence (% soft tissue) indicated no significant differences between experimental groups OA, OA/ZM, or OA/P/ZM relative to the CTRL (*p* = 0.341, *p* = 0.679, *p* = 0.982, respectively). However, qualitative analysis of the histological micrographs demonstrated advanced bone healing characterized by an abundance of nucleation sites for regeneration to occur in defects treated with OA relative to the CTRL. Bone overgrowth beyond the limits of defect borders was observed in groups treated OA/ZM and OA/P/ZM. In contrast to the treatment groups, minimal woven bone was visualized in the CTRL group.

**Conclusion:**

Compared to defects treated with porcine-derived particulate and barrier membrane (CTRL), defects filled with OA exhibited bone regeneration throughout the defect, with bone overgrowth when covered by a barrier membrane at 4-weeks *in vivo*. This suggests that the novel combination of AMP-2 and a bioceramic/synthetic polymer-based electrospun scaffold is a suitable candidate for GBR procedures, without a barrier membrane to secure its place within a defect.

## 1 Introduction

Caries, trauma, and periapical/periodontal disease are common indications for tooth extraction followed by endosteal implant placement (Broers et al., 2022). However, the tooth-dependent nature of the alveolar ridge makes it susceptible to significant resorption following tooth loss. Within 6 months of healing at an edentulous alveolar site, bone resorption has been estimated to occur between a range of 11%–22% and 29%–63% in the vertical and horizontal planes, respectively (Tan et al., 2012). Alveolar ridge preservation by guided bone regeneration (GBR) is a surgical procedure that encompasses placement of a barrier membrane with or without bone grafting material. An implant survival rate greater than 90% has been demonstrated when implants are placed in adjunct to a GBR procedure (Milinkovic and Cordaro, 2014). GBR has been shown to improve overall functional and aesthetic outcomes and reduce the need for future corrective procedures (Al Yafi et al., 2019). As a result, the market for dental bone graft materials is estimated to be worth over $450 million with projections to reach ∼$930 million in 2025 (Dental Bone Graft Substitute Market, 2020; Zhao et al., 2021). However, with the extent of commercially available bone grafting materials, clinicians often face overwhelming choices, particularly in the context of incomprehensive data with frequent reports of conflicting efficacy (Avila-Ortiz et al., 2019; Papageorgiou et al., 2016; Zampara et al., 2022).

A grafting material suitable for GBR should foster an environment that promotes osseointegration at the bone-graft interface and bone regeneration through osteoconduction and/or osteoinduction (Nayak et al., 2023b; Elgali et al., 2017). Traditionally, the placement of a barrier membrane minimizes rapid ingrowth of non-osteogenic cell lines, which would otherwise interfere with bone formation within the defect site (Kao and Scott, 2007; Wessing et al., 2018). While autologous bone grafts have long been considered the ‘*gold standard*’, there remains extensive debate over whether their benefits outweigh the drawbacks. This has included concerns with increased cost of care, prolonged operative time, extended postoperative pain, shorter resorption times of the material and increased risk of infection at the harvesting site (Papageorgiou et al., 2016; Neovius and Engstrand, 2010; Nguyen et al., 2018; Emara and Shah, 2021). Although allografts eliminate many of these shortcomings, they may transmit infection, elicit antigenic response, and are increasingly difficult to attain due to supply-demand volatility (Amini et al., 2012; Hill and Purtill, 2022).

Xenografts serve as viable alternatives to autografts, and allografts for GBR. While they are predominately deproteinized cancellous bovine bone matrices, porcine-derived xenografts have gained traction in recent years for use in GBR procedures (Bae et al., 2019; Bracey et al., 2018). Despite having high osteoconductivity and low immunogenicity, xenografts pose a risk of transmitting zoonotic infections while also requiring costly processing steps prior to implantation (Zhao et al., 2021; Ozkan et al., 2011; Meloni et al., 2018; Younes et al., 2019; Ibrahim et al., 2022). Synthetic bone grafts, or alloplasts, are particularly appealing as they are available in nearly unlimited quantities, may yield more consistent product quality and pose no risk of transmissible infection or other autograft-associated morbidities to the patient (Tomas et al., 2021). The advancement of innovative fabrication methods, such as three-dimensional (3D) printing, electrospinning, etc., have further facilitated the creation of high-fidelity, customizable, bioactive scaffolds for bone regenerative applications (Shen et al., 2020; Aytac et al., 2022). These scaffolds with tailorable physico-chemical properties have also enabled surgeons to restore the structure and function of large, complex-shaped bony defects which would be challenging to accomplish with autografts/allografts alone (Nayak et al., 2023a; Slavin et al., 2024). Recently, novel biomaterials such as graphene-based scaffolds, electrochemical sensing scaffolds, composite (polymer/polymer-bioceramic)-based bone scaffolds, among others, have been introduced that are that are better tailored to the intended *in vivo* environment, relative to the current standard-of-care grafting materials (Joyce et al., 2023; Xu et al., 2022; Zhao et al., 2024; Zhu et al., 2024).

Composite scaffolds composed of bioceramics (e.g., beta-tricalcium phosphate, calcium carbonate) and synthetic polymers (e.g., poly (L-lactide-co-glycolide)) offer both osteoconduction/osteoinduction and improved mechanical strength/degradation kinetics, respectively (Slavin et al., 2023; Khalaf et al., 2022; Watabe et al., 2021). Electrospinning techniques can be used to generate these composite scaffolds in the form of cotton-like fibers, which have been reported to possess a striking resemblance to the collagenous fibers of native extracellular matrix (Niemczyk-Soczynska et al., 2021). Fiber-based scaffolds possess a high surface-to-volume ratio, facilitating enhanced cellular attachment, thereby making them an attractive category of biomaterials for bony defect reconstruction in the oral cavity. Unlike the histological and clinical evidence of dehiscence and poor bone regeneration surrounding the use of immobilized particulate graft (which also require a membrane to remain in place for GBR) (Wessing et al., 2018), associated improvements in surface area owing to highly packed electrospun fibers can reduce the likelihood of the aforementioned drawbacks. Most importantly, in this context, a membrane may be an unnecessary adjunct to electrospun fibers for GBR, although to our knowledge this has yet to be proven (ORTHOReBIRTH, 2024).

On the other hand, innovative methods to further enhance tissue regeneration by the incorporation of stem cells and/or other osteoprogenitor cells into scaffolds, along with growth factors like platelet concentrates, bone morphogenetic protein (BMP) and other pharmacological agents, have been explored in preclinical studies (Ehlen et al., 2024a; Ehlen et al., 2024b). AMP-2 represents one such novel pharmacological agent, a variant of recombinant human BMP-2 (rhBMP-2), engineered to exhibit high affinity and specificity for the calcium phosphate component of a carrier matrix (Christou et al., 2024). The affinity leads to the retention of AMP-2 on the carrier at timescales that align more closely with those necessary for inducing new bone formation (Christou et al., 2024). This approach has been described to prevent the bolus release typically associated with rhBMP-2, minimizing unintended, off-target effects by promoting new bone formation at the precise anatomical location of scaffold placement (Christou et al., 2024).

**FIGURE 1:**
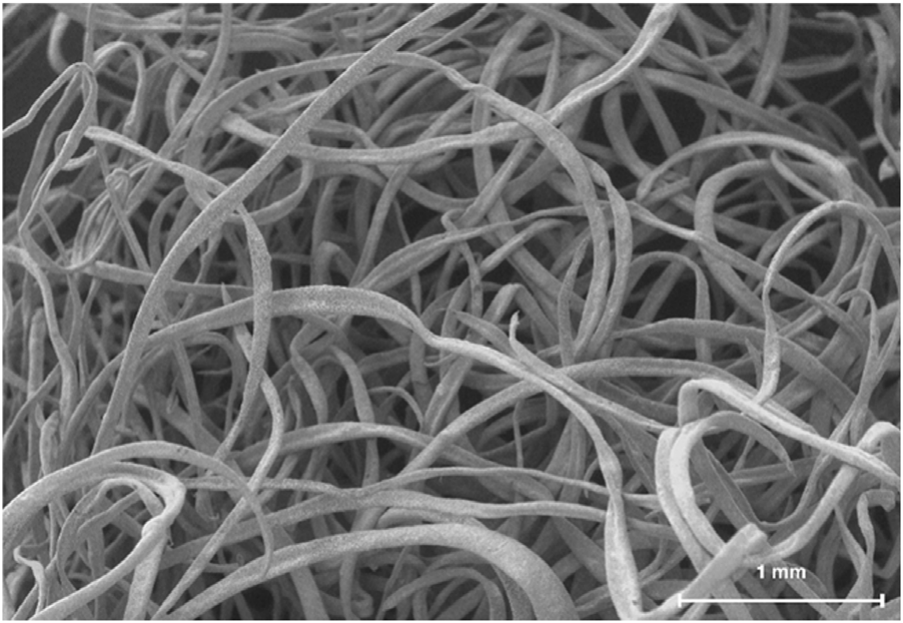
Representative scanning electron micrograph of OsteoAdapt, comprised of AMP-2 coated electrospun fibers.

OsteoAdapt (Theradaptive Inc, Frederick, MD, United States) (Figure 1), is a novel, bioactive fiber-based composite scaffold comprised of i) AMP-2 and ii) ReBOSSIS^®^ (ORTHOReBIRTH, Kanagawa, Japan), a 510-K cleared electrospun matrix containing beta-tricalcium phosphate (β-TCP), and poly (L-lactide-co-glycolide) (PLGA) (ORTHOReBIRTH, 2024). This study served as a proof-of-concept *in vivo* evaluation of the use of OsteoAdapt, with and without a porcine-derived barrier membrane and in combination with a porcine particulate, for GBR. The aim of this study was to utilize histologic, microtomographic, and histomorphometric analyses to evaluate the bone regenerative capacity of this novel electrospun composite scaffold in comparison to a predicate porcine-derived particulate and barrier membrane. Moreover, it sought to determine if OsteoAdapt would remain within the defect without a membrane in place, as this is not feasible with the particulate xenograft currently used in clinical practice (Osteogenics, 2024).

## 2 Materials and methods

### 2.1 Surgical protocol

The study was approved by an Institutional Animal Care and Use Committee (IACUC) committee, with all surgical procedures performed at an Association for Assessment and Accreditation of Laboratory Animal Care (AAALAC) approved facility. Surgery was performed on n = 4 adult (∼1.5 years of age) beagle dogs in good health. Animals underwent an acclimation period of 1 week prior to surgery. Subjects were fasted for 12 h ahead of anesthetization in preparation for the surgical procedure. Anesthesia was maintained throughout the duration of the surgery using isoflurane (0%–5%, in 100% O_2_ via inhalation, to effect), cefazolin (19–30 mg/kg, 1x, via intravenous injection (IV)), propofol (2–8 mg/kg, via IV, to effect), nitroglycerin (100–400 mcg, via intra-arterial injection (IA), to effect), and IsoVue contrast or equivalent (as needed, via IV).

**FIGURE 2:**
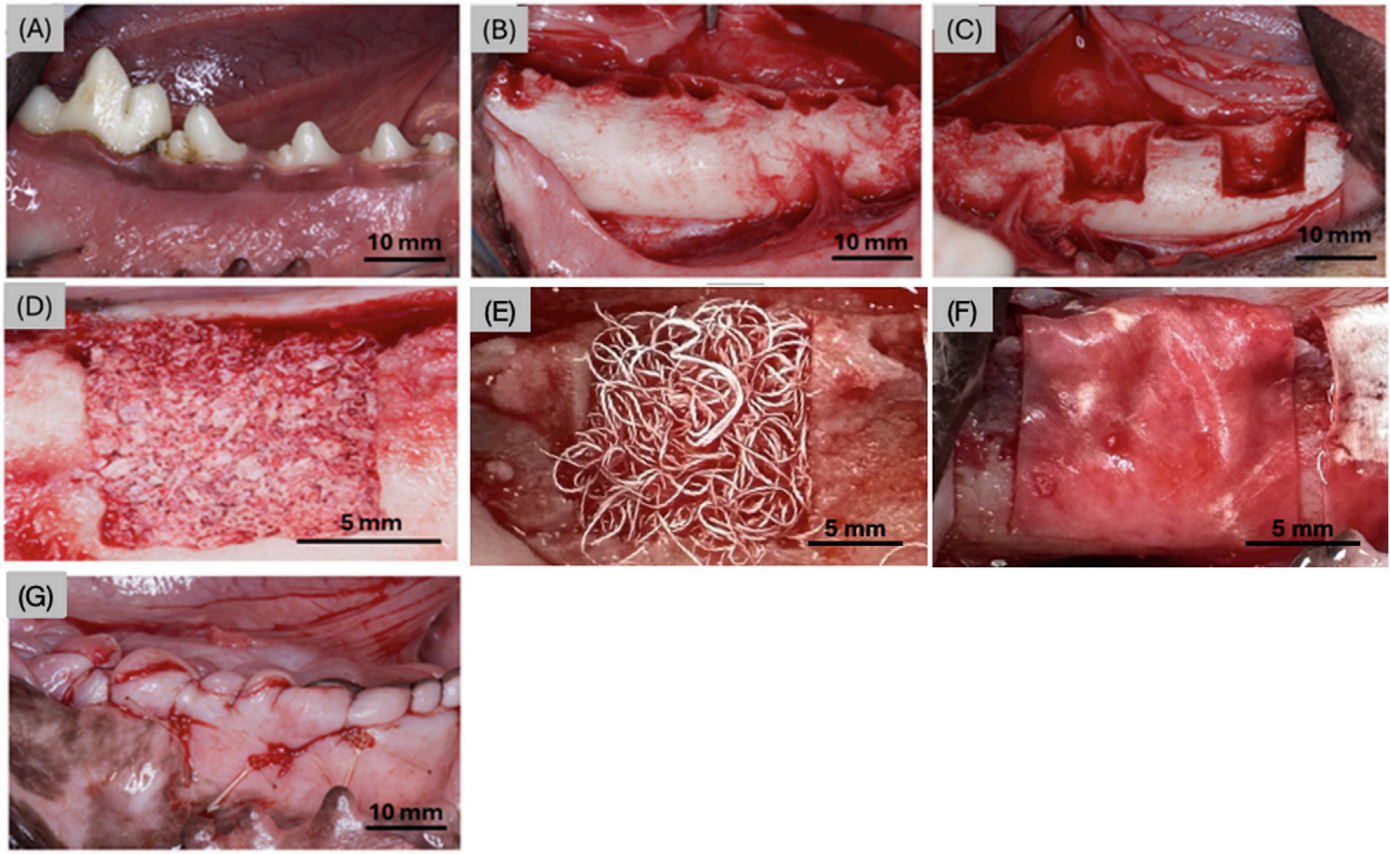
Digital images representing **(A)** the 4 premolars and molar prior to extraction, **(B)** the mandible post extraction, **(C)** two defects made in the anterior and posterior aspects of the mandible on both left and right sides (right side shown), representative defects filled with **(D)** Zcore™, **(E)** Osteodapt, **(F)** defect site covered with the Zmatrix™ porcine peritoneum collagen membrane (in the OA/ZM, OA/P/ZM and CTRL groups), and **(G)** surgical site post material placement, membrane and suturing.

During the first stage of the surgical procedure, the lower premolars (P1-P4) and first molar (M1) were extracted bilaterally (Figures 2A, B). Immediately following extraction, a mid-crestal incision was made to raise a full-thickness flap bilaterally, allowing for visualization of the exposed mandibular bone (Figure 2B). A low-speed cylindrical burr under copious irrigation was utilized to create semi-saddle, four-walled defects (n = 4 defects/dog) of 10 × 10 mm through the mandibular buccal plate with the lingual plate left intact (Figure 2C). Each defect was treated with one of the following: (i) OsteoAdapt (0.75 cc) without membrane (OA) (Theradaptive Inc, Frederick, MD, United States), (ii) OsteoAdapt (0.75 cc) with Zmatrix™ porcine peritoneum collagen membrane (15 mm × 20 mm, Regenity Biosciences, Paramus, NJ, United States) (OA/ZM), (iii) OsteoAdapt (0.5 cc) mixed with Zcore™ porcine xenograft (particle size: 0.25–1.0 mm) particulate (1 cc) (Regenity Biosciences, Paramus NJ, United States) and covered with Zmatrix™ porcine peritoneum collagen membrane (15 mm × 20 mm, Regenity Biosciences, Paramus, NJ, United States) (OA/P/ZM) or (iv) Zcore™ porcine xenograft particulate (2 cc) and Zmatrix™ porcine peritoneum collagen membrane (Positive Control - CTRL) (Figures 2D, E). The membranes were trimmed to shape and draped over the defect (Figure 2F) such that the defects were completely covered, in accordance with a previously published study (Bergamo et al., 2023). Flap advancement was achieved through periosteal release to allow for tension-free wound closure. Wounds were sutured using polytetrafluoroethylene (PTFE) sutures (Figure 2G). All experimental groups were nested within subjects and interpolated among defect sites to avoid site bias.

Post-operative pain was controlled with intramuscular injections of buprenorphine (0.05 mg/kg) and banamine (0.1 mg/kg) as needed. Prophylactic antibiotics (penicillin/streptomycin, 8–10 mg/kg and cephalexin, 3–5 mg/kg) were administered intramuscularly for 3 days following surgery. Animals were euthanized after 4-weeks post-operatively by means of anesthesia overdose using isoflurane (0%–5%, in 100% O_2_ via inhalation, to effect), propofol (2–8 mg/kg, via IV, to effect), heparin (20,000 IU, 1x, via IV), and potassium chloride (75–150 mg/kg, via IV, to effect). Mandibles, including graft/membranes, were harvested by sharp dissection (Bergamo et al., 2023; Kim et al., 2017; Gerard et al., 2006).

### 2.2 Three-dimensional (3D) volumetric reconstruction

Following euthanasia, samples were trimmed and stored in 70% ethanol. Scanning was performed using a micro-computed tomography (microCT) apparatus (μCT 40, Scanco Medical, Basserdorf, Switzerland) operated at 70 kV and 114 μA with a voxel resolution of 18 μm. The resulting files were exported in a Digital Imaging and Communications in Medicine (DICOM) format into a 3D reconstruction and segmentation software, Amira 6.3.2 (Thermo Fisher Scientific, Waltham, MA, United States). Within the software, specific materials and structures were differentiated based on contrast opacity including bone and graft. For accurate assessment of the 3D structures, a virtual slice technique was employed where a two-dimensional (2D) sagittal transection was acquired. This technique facilitated the adjustment of imaging parameters to minimize artifact and noise, thereby enhancing the visibility of newly regenerated bone. The differentiation of newly formed bone from pre-existing bone was by identifying variations in density (Hounsfield units), at the defect margins. The subsequent image segmentations were conducted by a single, trained and experienced investigator.

### 2.3 Histologic processing and analysis

The anterior and posterior defects were first separated prior to histological processing. *En bloc* samples were sequentially dehydrated in a series of ethanol solutions ranging from 70% to 100% followed by methyl salicylate. The samples were then embedded in methyl methacrylate (Bergamo et al., 2023; Bekisz et al., 2018; Bergamo et al., 2024). A low-speed precision diamond saw (Isomet 2000, Buehler Ltd., Lake Bluff, IL, United States) was utilized to section samples in the buccolingual plane of the mandible (∼300 μm thickness). This cutting direction was employed to visualize the entire mandibular cross-section on a single histological slide, encompassing the native lingual wall, defect space, and buccal mucosa. Each sample was wafered at the same region (middle of the alveolar bone defect, ∼5 mm from the anterior and/or posterior defect walls) to ensure standardization across the different samples. Individual tissue sections were then glued to acrylic plates with a cyanoacrylate adhesive (Henkel Loctite 408 adhesive, Dusseldorf, Germany) and left to set for 24 h. A series of silicon carbide (SiC) abrasive papers (400, 600, 800, and 1200 grit) were used to grind samples on a polishing wheel (Metaserv 3000, Buehler Ltd., Lake Bluff, IL, United States) under irrigation until all samples reached a final thickness of ∼100 μm. Slides were then polished using a microfiber cloth and 1.0 µm micro-polish alumina particle suspension (Buehler Ltd., Lake Bluff, IL, United States). After polishing, slides were stained with Stevenel’s Blue and Van Gieson’s Picro Fuchsin (SVG) stain in preparation for digital scanning. Stevenel’s blue stained cells and extracelluar structures in a gradation of blue tones. Van Gieson’s picro fuchsin, stained collagen fibers green or green-blue; bone in red, orange or purple; and muscle fibers in blue to blue-green. An automated slide scanning system (Aperio CS2, Leica Biosystems, Wetzlar, Germany) was used to capture high-resolution digital images which were subsequently analyzed by a trained investigator.

### 2.4 Histomorphometric analysis

The high-resolution digital scans were imported into Photoshop (Adobe, San Jose, CA, United States) to quantify bone (bone %), soft tissue (soft tissue %) and in certain samples/groups amount of porcine graft (particulate graft %). All slides were stained with Stevenel’s Blue and Van Giesons Picro Fuschin which provided sufficient differentiation between the different tissue and cell types. For each scan, the region of interest (ROI) was identified using the defect margins clearly visible on the histomicrographs and were manually delineated using color selectors to highlight individual tissue and graft (when present), by a single, trained investigator to ensure consistency between samples. A customized computer software (JV Analysis, Biomaterials Division, New York University, NY, United States; Department of Biochemistry and Molecular Biology, University of Miami, FL, United States) allowed for the quantification of the total area of each of the colored regions reported as a percentage.

### 2.5 Statistical analysis

The sample size was determined by power analysis, to have a statistical power greater than 0.8, and type I error frequency (α) of 0.05. Based on statistical power analysis, a minimum of n = 16 defects were required for analysis of outcome variables. To minimize the number of animals used for this study, the experimental groups were nested within the animals (n = 4 defects per dog) for a total of 16 defects. Statistical evaluation of histomorphometric data (bone (%), soft tissue (%), and porcine graft (%) was performed using a mixed model analysis of variance and least significant difference (LSD) *post hoc* analyses (IBM SPSS v29, IBM Corp., Armonk, NY, USA). All numerical data are depicted as mean values with corresponding 95% confidence interval values (mean ± 95% CI) and *p* ≤ 0.05 denotes statistical significance.

## 3 Results

No complications, including infection, atypical inflammation, or bleeding, were observed during the surgical procedure or at any of the subsequent post-operative follow-ups.

### 3.1 Qualitative volumetric reconstruction

**FIGURE 3:**
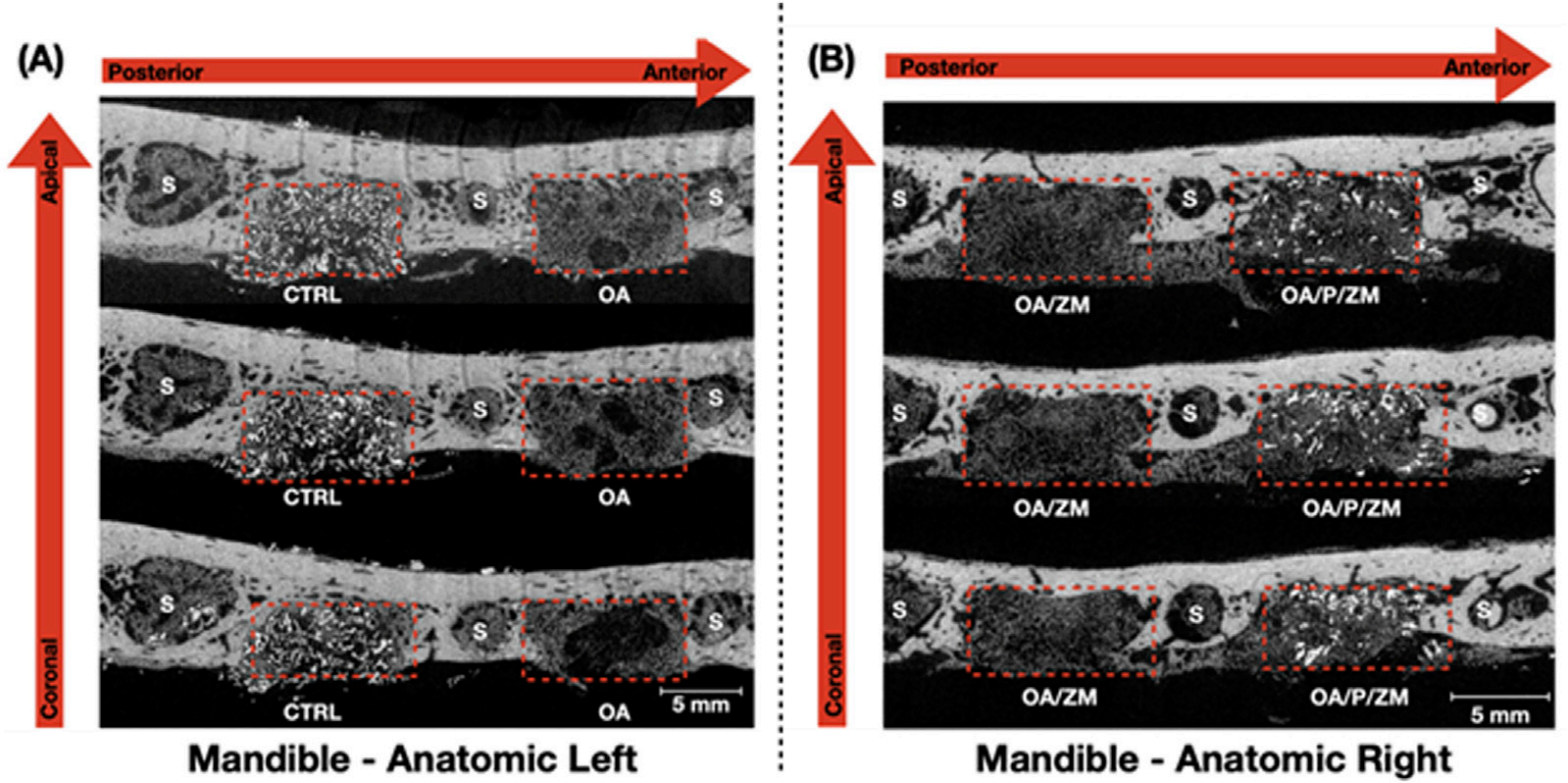
Representative grayscale microCT images (obtained along the transverse plane) of the anatomic **(A)** left and **(B)** right side of *en bloc* mandible samples showcasing the defects (dashed red boxes) filled with the different grafting materials used in this study. The red arrows indicate the sequence of microCT images presented from the coronal/apical and the anterior/posterior aspects. S = Sockets after teeth extraction.

**FIGURE 4:**
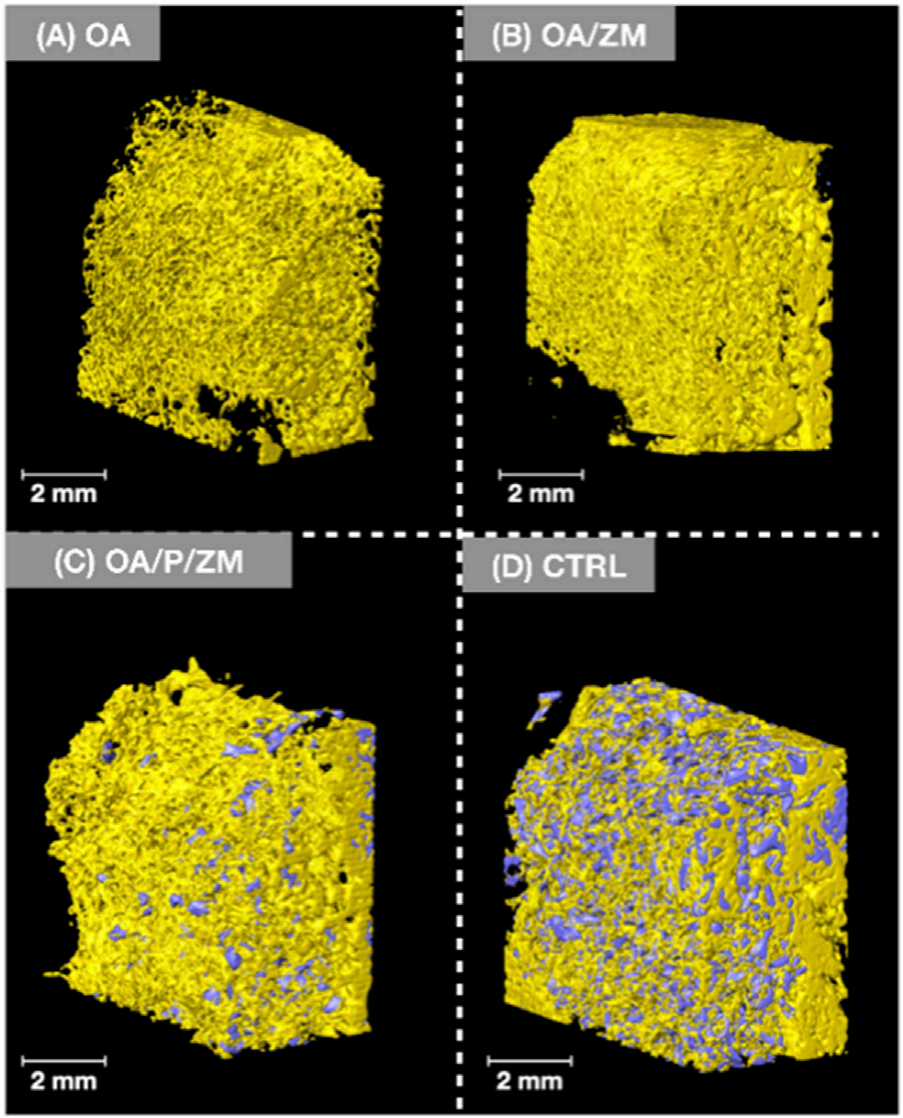
Volumetric reconstruction of defects treated with **(A)** OA, **(B)** OA/ZM, **(C)** OA/P/ZM, **(D)** CTRL. Regenerated bone is represented in yellow and particulate graft in purple. Images shown are representative and are oriented in the buccal-lingual (BL) direction.

It is pertinent to mention that newly formed bone exhibits a greater density and radio-opacity compared to the electrospun fibers and/or membrane (Zmatrix™) used within this study - which present the lowest density. In addition, due to overlapping thresholds between the electrospun fibers/membranes and background noise, quantification of percent bone versus material relative to the total volume segmentation was skewed. As such, volumetric reconstruction was elected to be utilized for qualitative analysis. 3D volumetric reconstruction of defect sites after 4-weeks *in vivo* demonstrated the presence of regenerated bone among all groups. Newly regenerated bone (Figures 3, 4A–D: yellow) within the center and at the defect borders was distinguishable from native bone due to its spongy appearance and corresponding lower radiopacity (Figure 3). The porcine particulate graft material (Figures 3, 4C-D: purple) was unevenly dispersed among regenerating bone in defects treated with OA/P/ZM and CTRL. The cotton-like, electrospun fibers of OsteoAdapt in OA, OA/ZM, and OA/P/ZM groups were not easily delineated from noise, and thus were not represented in the volumetric reconstruction.

### 3.2 Histological analysis

**FIGURE 5:**
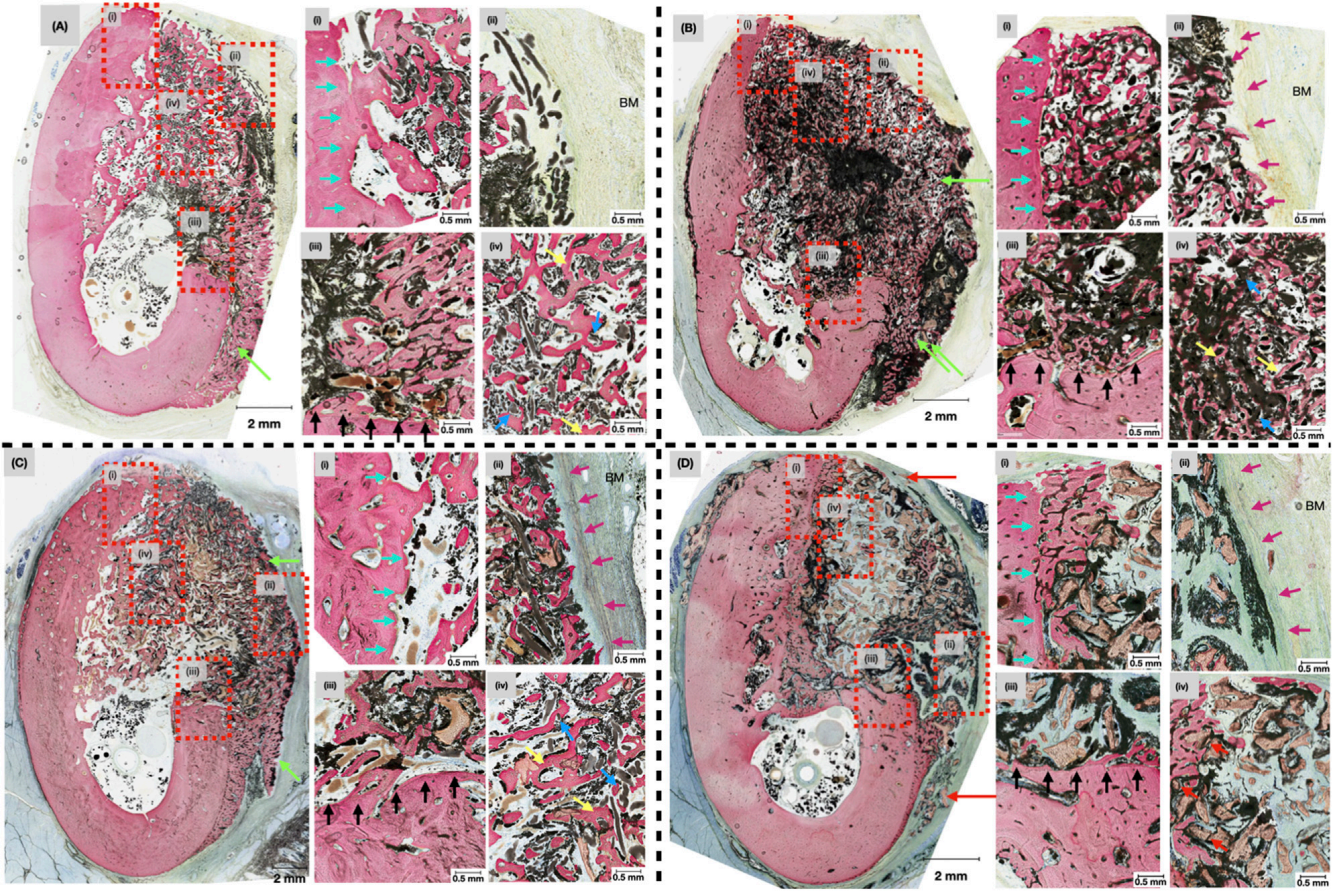
Representative histomicrographs of mandibular defects treated with **(A)** OA, **(B)** OA/ZM, **(C)** OA/P/ZM, **(D)** CTRL, after 4-weeks *in vivo* at low magnification. Green arrows indicate areas of bone overgrowth in the lateral and inferior regions of the buccal plate. Dashed red boxes highlight approximate locations of the high-magnification inserts (i-iv). Blue arrows indicate the presence of electrospun fibers with surrounding regenerated bone. Yellow arrows represent formation of a primitive trabecular network. Red arrows demonstrate areas of woven bone surrounding particulate graft (in the CTRL group), cyan arrows delineate the lingual wall of the defects, purple arrows show the area of membrane placement in proximity to the buccal mucosa (BM) in the OA/ZM, OA/P/ZM, CTRL groups, and black arrows highlight the apical/inferior wall of the induced bone defects.

At 4-weeks *in vivo,* qualitative evaluation of histological micrographs revealed the presence of bone regeneration among all groups (Figure 5). The quality, under high magnification, of regenerated bone in defects treated with OsteoAdapt with or without membrane (OA or OA/ZM) and particulate (OA/P/ZM) resembled a trabecular network (Figures 5A–C, yellow arrows). Within all treatment groups, the cotton-like fibers of OsteoAdapt were found to be engrossed within newly formed bone, providing an abundance of nucleation sites for regeneration to occur (Figures 5A–C, blue arrows). Despite the absence of a membrane in the OA group, electrospun fibers remained tightly packed within the boundaries of the defect in all subjects (Figure 5A). Near the native bone walls, defects treated with OA presented Haversian-like systems with lamellar reorganization, demonstrating the progressive maturation of newly formed bone. Additionally, bone regeneration was confined to the defect margin, with minimal overgrowth inferiorly at the region of the mandibular buccal plate, thereby preserving the native anatomic structure of the mandibular alveolar ridge (Figure 5A, green arrows). By contrast experimental groups treated with OsteoAdapt and covered by the porcine membrane (OA/ZM and OA/P/ZM) displayed bone overgrowth beyond the defect margins both in the inferior and lateral directions (Figures 5B, C, green arrows). The bone growth over the membrane was characterized by a pattern of immature woven bone. Nonetheless, these groups presented direct contact between membrane and bone (Figures 5B, C, purple arrows), indicating the osteoconductive potential of the membrane, and regions with soft tissue abutment. On the other hand, the membrane/soft tissue interface was primarily composed off fibrovascular connective tissue. Loose particulate graft was visible in defects treated with OA/P/ZM and CTRL groups. More specifically, in the CTRL group, bone appeared woven in nature and surrounded the graft material primarily in the defect’s periphery and only sparsely in the defect center (Figure 5D, red arrows).

### 3.3 Histomorphometric analysis

**FIGURE 6:**
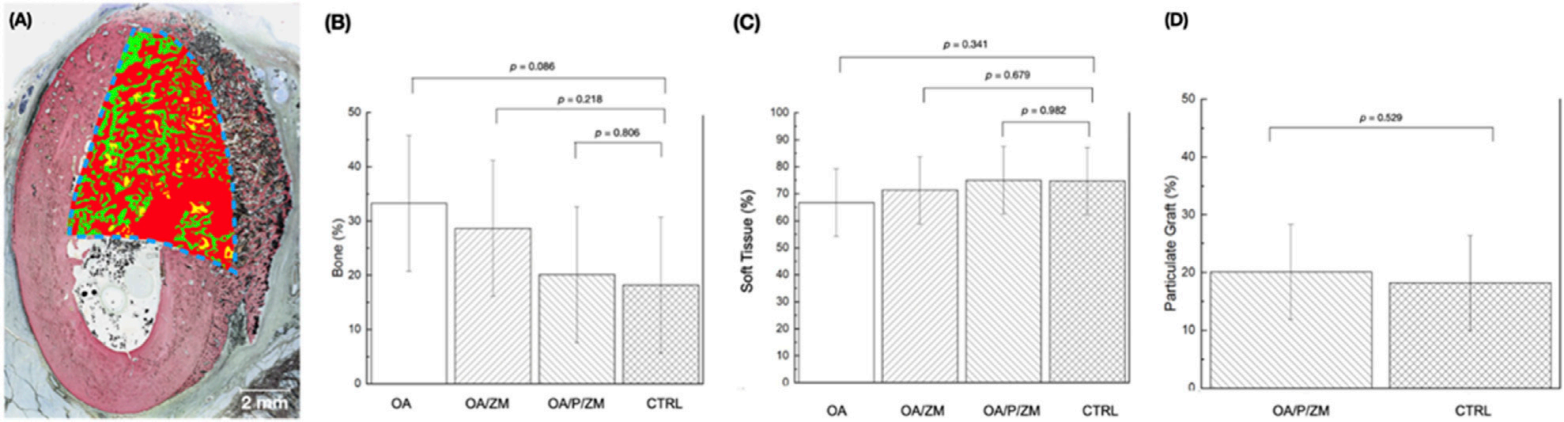
**(A)** Representative histomorphometric analysis of the region of interest (dashed blue lines) superimposed upon the histological overview depicting bone in green, particulate graft in yellow, and soft tissue in red. Histomorphometric analysis depicting **(B)** bone (%) and **(C)** soft tissue (%) within defects treated with OA, OA/ZM, OA/P/ZM, and CTRL. In addition, **(D)** particulate graft (%) was determined for groups OA/P/ZM and CTRL. All values are presented as means with corresponding 95% confidence interval values. *p* ≤ 0.05 is statistically significant.

Quantitative measurements of bone regeneration (% bone) within the ROI (Figure 6A) revealed no significant differences between OsteoAdapt without membrane (OA) (33.26 ± 15.98), OsteoAdapt with porcine membrane (OA/ZM) (28.64% ± 13.48), or OsteoAdapt mixed with particulate xenograft and porcine membrane (OA/P/ZM) (20.11% ± 3.35) in comparison to the porcine xenograft particulate and porcine membrane (CTRL) (18.18% ± 8.88) at 4-weeks *in vivo* (*p* = 0.086, *p* = 0.218, and *p* = 0.806, respectively) (Figure 6B). It is important to highlight that the quantity (mean) of percent regenerated bone decreased linearly across groups, with OA exhibiting the greatest amount and the CTRL group, the least. Statistical evaluation of soft tissue presence (% soft tissue) indicated no significant differences between experimental groups (66.74% ± 15.98 [OA], 71.36% ± 13.48 [OA/ZM] and 75.00% ± 2.58 [OA/P/ZM]) and the CTRL (74.69% ± 9.11) (*p* = 0.341, *p* = 0.679, *p* = 0.982, respectively) (Figure 6C). Lastly, the amount of porcine graft (% particulate graft) present at 4-weeks *in vivo* in defect sites belonging to OA/P/ZM (20.11% ± 3.35) and CTRL groups (18.18% ± 8.88) was also statistically homogeneous (*p* = 0.529) (Figure 6D).

## 4 Discussion

Given its striking similarity to humans, the canine mandible has been described as an excellent pre-clinical model to evaluate guided bone regeneration for clinical translation (Zhang et al., 2021). Alloplasts continue to gain traction amongst clinicians and patients as a non-autograft alternative for GBR (Almutairi, 2019; Assari et al., 2022). Advancements in guided bone regeneration (GBR) have led to the development of fiber-based composite scaffolds that can be produced through techniques like electrospinning. Associated improvements in surface area seen with highly packed electrospun fibers reduces the likelihood of dehiscence and poor bone regeneration often associated with poorly immobilized, commercially available, traditional particulate graft, which require a membrane to remain in place. In this context, a membrane may be an unnecessary adjunct to electrospun fibers for GBR. The present study sought to compare the *in vivo* performance of a novel, electrospun alloplastic composite scaffold, OsteoAdapt, with or without the addition of a barrier membrane and porcine-derived xenograft, to regenerate hard tissue defects in a beagle mandibular model. Quantitative histomorphometric analyses revealed no significant differences between experimental groups with respect to amount of bone regenerated or soft tissue presence within treated defects. While differences in regenerated bone were not significant, a distinctive trend was noted. The greatest amount of regenerated bone was observed in the OA group; and, quantity of regenerated bone decreased linearly across the remaining OA/ZM, OA/P/ZM and CTRL groups. Further, bone remodeling was appreciably more advanced in treatment groups containing OsteoAdapt relative to the xenograft CTRL group, as a primitive trabecular network was established at 4-weeks *in vivo*.

ReBOSSIS^®^ (ORTHOReBIRTH, Kanagawa, Japan), is an electrospun matrix containing 70% β-TCP and 30% PLGA, augmented with AMP-2 protein bound to the fibers. The advanced bone healing observed in groups treated with OsteoAdapt is postulated to be attributed both to its unique build and composition. ReBOSSIS^®^ has received 510(k) clearance by the FDA to act as a bone void filler, deeming it substantially equivalent to a legally marketed predicate device for use in the extremities, pelvis, posterolateral spine, and intervertebral disc space (Food and Drug Administration, 2024). This is substantiated by large preclinical studies, one of which found that the use of ReBOSSIS^®^ in combination with bone marrow aspirate and iliac crest autograft demonstrated similar spinal fusion rates in male rabbits at 8- and 12-weeks compared to the autograft alone (Nepola et al., 2019). These findings, at minimum, implicate that less autograft would be required from the patient with likely associated reductions in post-operative pain and extended operative time, as the study did not examine the fusion rate when using ReBOSSIS^®^ alone (Nepola et al., 2019).

With respect to the use of ReBOSSIS^®^ for mandibular bone regeneration, a recent study by Ramanathan et al. utilized ReBOSSIS^®^ to repair critically-sized mandibular defects in comparison to a pure β-TCP block without membranes (Ramanathan et al., 2023). After 12-weeks *in vivo,* expression of genes responsible for osteoblast differentiation remained elevated, suggesting continued bone regeneration at extended time points in both groups. However, a significantly greater bone volume to total volume (BV/TV) ratio was observed in defects treated with pure β-TCP. This was hypothesized to be partially due to enhanced surface roughness of the β-TCP block, allowing for increased osteogenic cell attachment and proliferation (Ramanathan et al., 2023). The cotton-like fibers generated from the electrospinning manufacturing process created an easily packable (and maintainable in place) material into large or uneven bone defects which could otherwise be unachievable with loose particulate grafts (Ramanathan et al., 2023). This ease-of-handling benefit was corroborated by our study which found that the electrospun scaffold remained packed within the defect site even with the absence of a membrane after 4-weeks *in vivo*. It is important to stress that material migration outside of the defect’s bounds did not occur, even in the setting of the animals returning to regular diets and physical activity post-operatively. In an effort to continue to improve the regenerative capacity of ReBOSSIS^®^ and other electrospun composite scaffolds, the addition of coatings intended to promote osteogenic cell migration and adhesion has recently been explored (Watabe et al., 2021; Briggs et al., 2015; Obata et al., 2020).

The addition of an AMP2 coating to ReBOSSIS^®^ - a proprietary variant of rh-BMP2 developed by Theradaptive Inc., provides the scaffold additional osteoinductive properties (Theradaptive, 2024). The AMP-2 recombinant variant binds more tightly to its scaffold in contrast to rh-BMP2 which does not exhibit significant binding to its carrier. Consequently, AMP2 should allow for precise and controlled bone regeneration, making it a safer alternative to rh-BMP2 - without the associated risk of ectopic bone reformation, and potential for improved patient healing outcomes (Theradaptive, 2024; Kinoshita and Maeda, 2013; Ramly et al., 2019; James et al., 2016). While studies have yet to utilize OsteoAdapt for the purposes of mandibular bone regeneration, preliminary results of several *in vivo* studies have demonstrated its superior bone regenerative capacity when compared to the standard-of-care autograft (Theradaptive, 2024; Douglas et al., 2023). To the best of the authors’ knowledge this is the first *in vivo* study to examine OsteoAdapt for the purposes of alveolar ridge augmentation. In the current study, when comparing the bone regenerative capacity of OA alone versus OA/ZM, or the OA/P/ZM, bone overgrowth at the mandibular buccal plate was exhibited predominantly in the OA/ZM and the OA/P/ZM groups. Although bone overgrowth was visualized in these groups, possibly alluding to a native regenerative response, percent bone remained highest within defects treated with OsteoAdapt with the absence of a membrane.

As the barrier membrane serves as one of the key principles of GBR, the ability of OsteoAdapt to restore the anatomical contour as early as 4-weeks *in vivo* without the occlusive membrane warrants future investigative studies. It has been well-documented that collagenous membranes begin to exhibit early signs of degradation after 2–4 weeks *in vivo* with resulting reductions in both their barrier properties and space maintenance abilities (Calciolari et al., 2018; Di Raimondo et al., 2021). However, it is important to note that the basal lamina, a component of the extracellular matrix which forms a barrier between the defect space and soft tissue, begins to form as early as 2-weeks (Al-Kattan et al., 2017). This may explain why no significant differences were detected between the three different groups that comprised of OsteoAdapt. Furthermore, it is plausible that the use of a non-collagenous membrane to cover OsteoAdapt, with improved physical properties and degradation, may remain intact long enough to allow for significant differences in bone regenerative outcomes compared to the use of OsteoAdapt alone, necessitating future studies to conclusively prove this phenomenon (Calciolari et al., 2018; Di Raimondo et al., 2021).

At 4-weeks *in vivo*, mandibular defects treated with OsteoAdapt exhibited bone regeneration throughout the defect, with bone overgrowth when covered by a barrier membrane. Additionally, advanced bone quality and structure was evident in these groups relative to the porcine xenograft and membrane group (CTRL). Of utmost significance, the electrospun fibers were easily packed to fill the entire defect and remained in place without the need for a membrane, questioning the once assumed necessity of membrane use in GBR procedures. However, it is essential to acknowledge the limitations of this study. First, this study is an evaluation of bone regeneration exclusively at the 4-week time point. Notably, this timepoint demonstrated favorable outcomes in bone regeneration, which may be attributed to the incorporation of the AMP-2 surface coating. To our knowledge, the current study focuses on a novel material that has not been evaluated in the literature for GBR and aimed to serve as a baseline for future research on similar topics. As such, the promising results at this stage in the healing cascade suggest that future studies should explore shorter (2 weeks) and longer (8 weeks) timepoints to provide an objective measurement of the regenerative process through techniques like bone mineral density quantification (by dual energy X-ray absorptiometry), and scaffold/graft degradation. Next, while OA and CTRL groups quantitatively presented homogenous bone regeneration within the defect sites at 4 weeks, an increase in sample size could potentially reveal statistically significant differences. Furthermore, the utilization of a synthetic biodegradable polymer membrane with OsteoAdapt in comparison to a collagen membrane should be investigated to identify if differences in their physiochemical properties translate to changes in bone regeneration. Finally, although previous studies have supported the contribution of AMP-2 to enhance bone formation, it is clinically pertinent to state that pharmacological-based approaches can pose regulatory obstacles owing to the potential to elicit host immune responses, necessitating clinical trials. For this reason, OsteoAdapt was granted Breakthrough Medical Device designation by the FDA, and clinical trials are currently underway (Medicine USNLo, 2023).

## Data Availability

The raw data supporting the conclusions of this article will be made available by the authors, without undue reservation.
